# Design and additive manufacturing of a lightweight aerospace electric actuator

**DOI:** 10.12688/openreseurope.17752.1

**Published:** 2024-06-21

**Authors:** Borja Lizarribar Carrillo, Borja Prieto, Miguel Martínez-Iturralde, Javier García Goikoetxea, Sergio Montes, Ekain San José

**Affiliations:** 1 CEIT-Basque Research and Technology Alliance (BRTA), Manuel Lardizabal 15, Donostia / San Sebastián, 20018, Spain; 2 Universidad de Navarra, Tecnun,, Manuel Lardizabal 13, Donostia / San Sebastián, 20018, Spain; 3 Egile Mechanics S.L., Polígono Industrial Kurutz-Gain, 12, Mendaro, 20850, Spain

**Keywords:** Electrical Machine, Additive Manufacturing, Topology Optimization, Laser Powder Bed Fusion, Selective Laser Melting, Electric Actuator, Electric Motor, Permanent Magnet Machine

## Abstract

**Background:**

The ambitious electrification targets set for the aeronautical sector are leading to a thorough research into improving the performance of different electromechanical components. In this regard, Additive Manufacturing is gaining strength due to the positive physical properties of the processed parts and the freedom in manufacturable geometries.

**Methods:**

Thus, this article presents the design of an electric actuator for an aerospace active sidestick in which Additive Manufacturing is used with the aim of minimising the weight and power consumption of the device. The electromagnetic design of the actuator is detailed, considering 8 different permanent magnet machine topologies, and a mechanical design applying Topology Optimisation to reduce the overall weight of the component is carried out.

**Results:**

Three prototypes involving the rotor, the stator and the casing are manufactured via Laser Powder Bed Fusion in stainless steel and Permendur (Fe _49_Co _49_V _2_) and the corresponding actuators are experimentally tested, showing a great agreement between tests and simulations and excellent repeatability in the electromagnetic behaviour of the prototypes.

**Conclusions:**

The research results highlight the great potential of Additive Manufacturing to manufacture functional electrical machine components.

## Plain language summary

In order to analyse the potential benefits of using additive manufacturing in the production of electrical machines, this work carries out the mechanical and electromagnetic design of an electric motor, taking into account the geometrical advantages that additive manufacturing allows for the electromagnetic and mechanical design. Finally, three prototypes are manufactured and tested, showing good agreement between simulation and test results.

## 1 Introduction

The global objectives of mitigating fuel consumption and reducing *CO*_2_ and *NO _x_* emissions are leading to a profound interest in the electrification of components that were not traditionally electric. One example of this is the Aeronautical sector, in which the growing emphasis on electrification is coupled with a continuous advancement and integration of sophisticated lightweight components ^1– 3^. To date, the focus has been placed mainly on reducing the mass of mechanical functional components via costly iterative mechanical design. However, the innovative designs that can best fulfill the weight reduction goal are typically difficult to produce using traditional manufacturing methods.

In this context, Topology Optimization (TO) and Additive Manufacturing (AM) confer substantial advantages by optimally distributing material within the given design space and creating lightweight and structurally optimized components, without compromising strength. Moreover, this approach enables rapid prototyping and customization, with minimum material waste, fostering innovation and agility in responding to evolving industry requirements.

Regarding TO and AM of electrical machines, the manufacture of complete functional equipment, which meets the requirements of a given application, has been little explored ^4– 6^. Research to date has mainly focused on the processing of different metallic alloys (e.g. Ti-based ^7– 9^, Cu-based ^10– 12^, Al-based ^13, 14^, steel-based ^15^, etc.) and concept validators involving a single component, such as toroids ^16– 18^ and coils ^19– 22^. With regard to TO, previous work tends to focus on algorithm development rather than on the actual application requirements ^23– 27^.

Owing to the above, this article focuses on the use of AM and TO for the design of an electric actuator that fully meets certain design requirements, with the focus on obtaining multifunctional components with dual magnetic-structural function. Additionally, the option of adding features that provide benefits to the design that are impossible to achieve with conventional manufacturing by lamination and machining is explored.

## 2 Methods

### 2.1 Requirements of the actuator application

**Figure 1.**
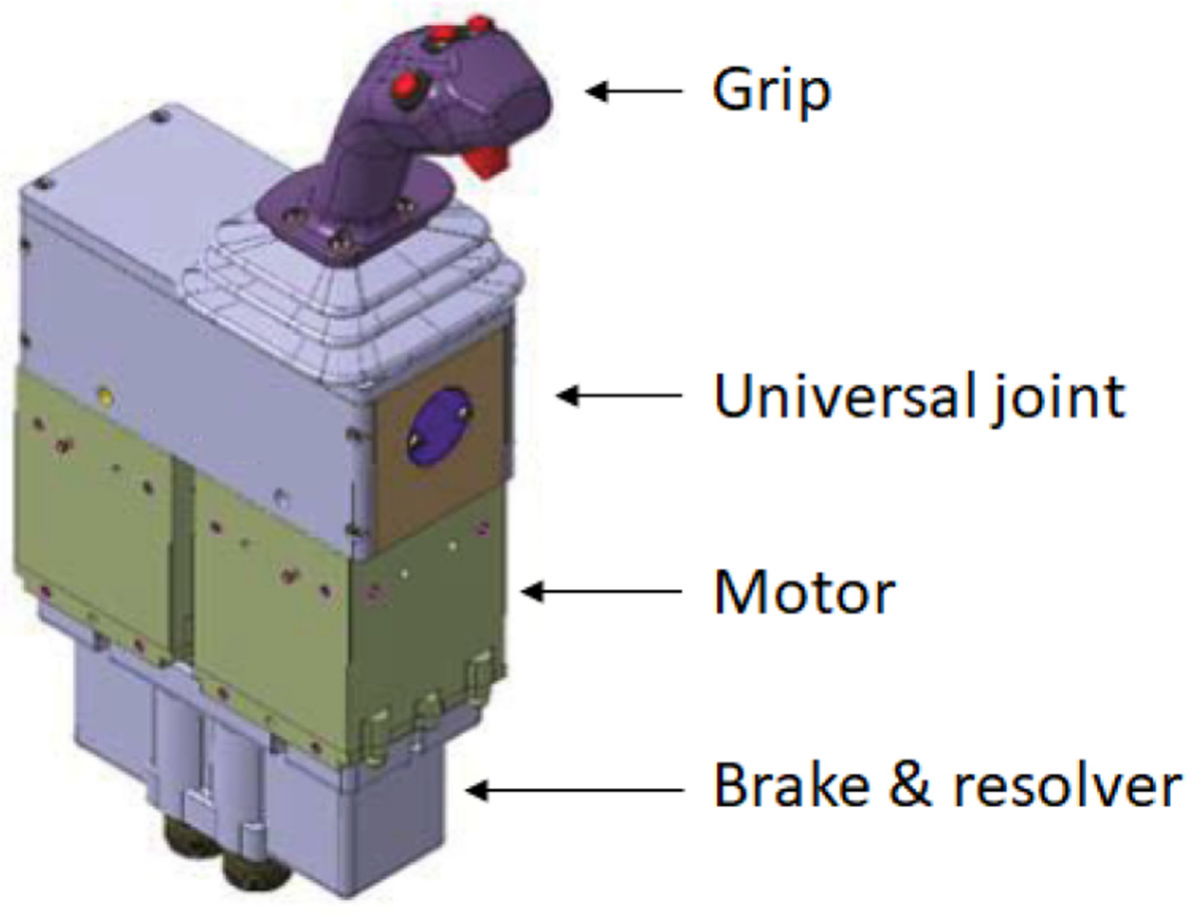
Concept for the active sidestick system (Smart Active Inceptor by Safran Electronics & Defense) ^28^.

The designed electric actuator is part of an active sidestick system which is illustrated in Figure 1. With the help of two electric motors coupled to an universal joint, controlled force-feedback is provided to the grip in 2-axis in a quasi-static way (i.e. the angular speed of the electric motors is typically < 1 rad/s). The performance requirements for each of the electric motors are gathered in Table 1.

**Table 1.** Main requirements for the designed actuator.

Requirements	
**Envelope**	*100 x 100 x 100 mm*
**Torque**	*8 N·m*
**Max. power consumption** **(stall conditions)**	*150 W*
**Max. temperature after** **120 s operation (stall)**	*120 ºC*
**Weight goal**	*≤ 3 kg*

For the case studied, involving a nearly zero speed application, the AM of soft magnetic materials is highly convenient. In conventional AC motors, working typically at 50/60 Hz, the variable magnetic field in the soft-magnetic material parts and the associated eddy currents lead to considerable power losses in these components, if manufactured as solid blocks. To reduce eddy current losses, rotors and stators of AC machines are traditionally manufactured by die-cutting and stacking insulated thin silicon steel laminations. With regard to components manufactured by additive approaches, several strategies have been explored already in the literature in which semi-lamination strategies are applied to mitigate these losses ^29– 31^. In the present work, due to the extremely low speed and frequency of the application at hand, no lamination or eddy current loss reduction strategy for the soft-magnetic parts has been considered. The material selected for these components is Permendur, an iron-cobalt-vanadium alloy. Details regarding material assessment and optimization of process parameters, including thermal treatment, can be found in 32.

Additive manufacturing of hard-magnetic materials has been discarded within the project, as for now, the magnetic performance (i.e. coercivity, remanence, energy product) of AM hard-magnetic materials is far from that of conventional sintered rare-earth magnets ^33– 36^. Furthermore, the little space available and the high number of turns derived from the low speed has led to discard the AM of electrical conductors. Conversely, the AM of structural parts in 316L stainless steel has been investigated.

### 2.2 Actuator topology selection

**Figure 2.**
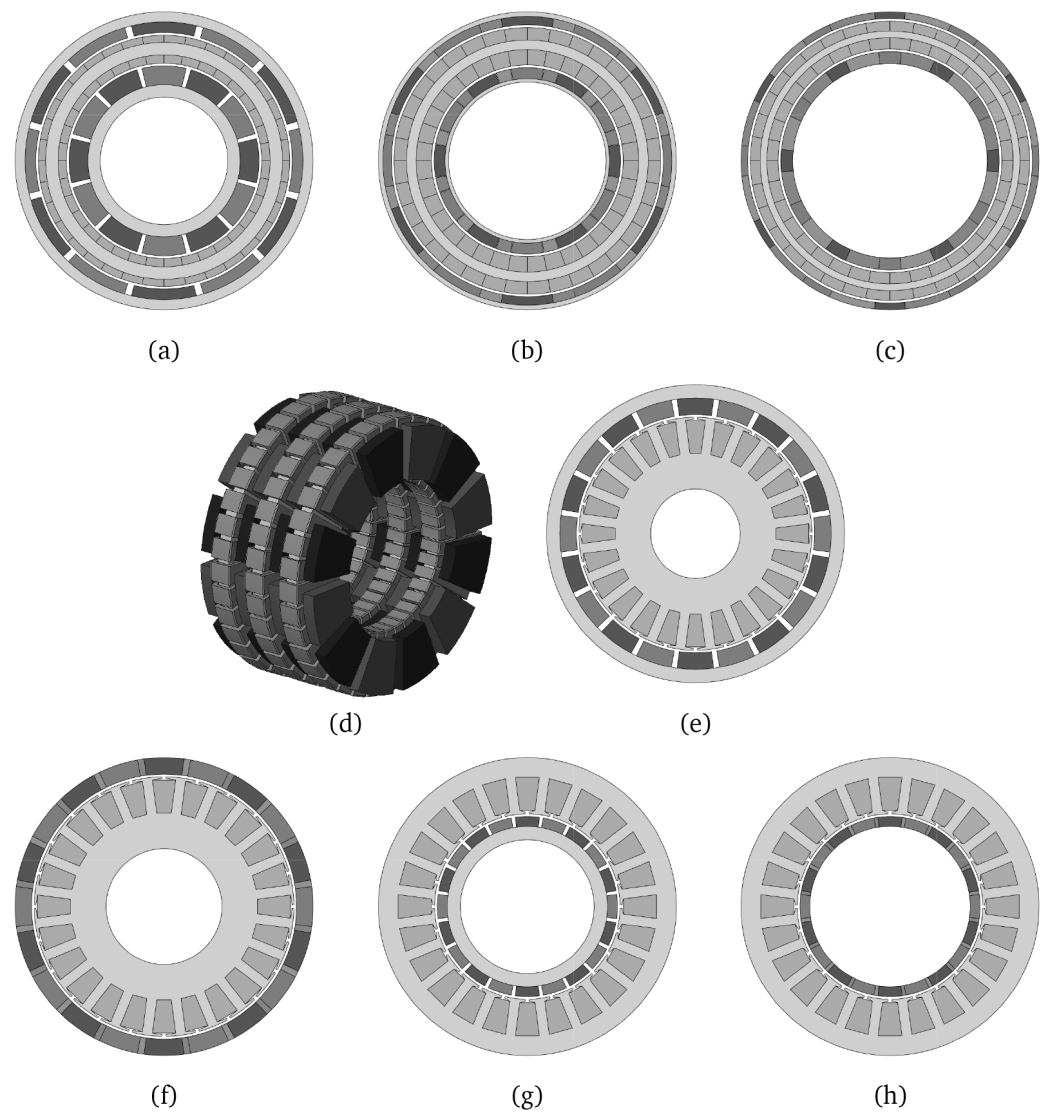
Analysed motor configurations ( **a**) radial flux slotless double rotor, ( **b**) radial flux slotless double rotor with Halbach magnets, ( **c**) radial flux slotless double rotor yokeless Halbach, ( **d**) three-stage axial flux modified as in 38, ( **e**) radial flux outer rotor, ( **f**) radial flux outer rotor Halbach, ( **g**) radial flux inner rotor and ( **h**) radial flux inner rotor Halbach.

**Figure 3.**
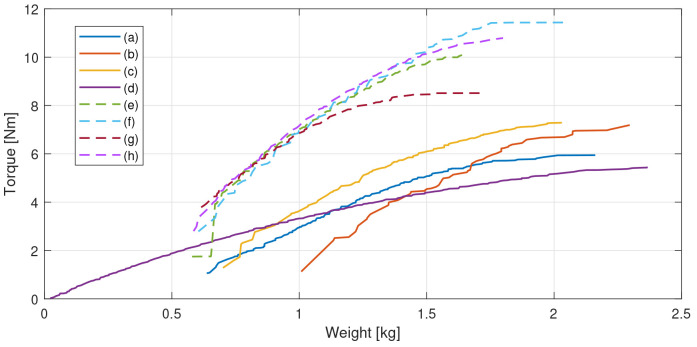
Pareto fronts for the assessed configurations, ( **a**) radial flux slotless double rotor, ( **b**) radial flux slotless double rotor with Halbach magnets, ( **c**) radial flux slotless double rotor yokeless Halbach, ( **d**) three-stage axial flux modified as in 38, ( **e**) radial flux outer rotor, ( **f**) radial flux outer rotor Halbach, ( **g**) radial flux inner rotor and ( **h**) radial flux inner rotor Halbach.

To explore the possibilities enabled by AM and to select the preliminary actuator geometry with the highest torque density, 8 different machine topologies have been analyzed for a maximum stack length of 55 mm. The studied topologies, which are illustrated in Figure 2, correspond to 6-phase AC permanent magnet motor configurations for the highest torque density. All the depicted shapes have been optimised parametrically in terms of FEA, varying the geometry for each individual via the evolutionary algorithm MODE (Multi Objective Differential Evolution) ^37^. The objectives to be optimised have been the weight of the active parts and the electromagnetic torque for the maximum stall power defined in Table 1. The electromagnetic performance has been computed via magnetostatic 2D FE simulations in the software FEMM ^®^ by considering the maximum allowable temperature for the magnets and the windings. The DC resistance of the stator has been computed analytically taking into account the mean turn length. The input current has been set for the maximum DC power consumption conditions. The materials considered in the electromagnetic simulation are gathered in Table 2. The results in form of Pareto front obtained for the optimisation of each electrical machine configuration are shown in Figure 3.

**Table 2.** Materials used in the electromagnetic FEM simulation.

Component	Material
Rotor	Permendur (Fe _49_Co _49_V _2_)
Stator	Permendur (Fe _49_Co _49_V _2_)
Permanent Magnets	Sm _2_Co _17_ (YXG-32 grade)
Electrical conductors	Enamelled copper wire

From Figure 3, it can be noticed that the motor topologies that are able to achieve the 8 N·m requirement with a DC power consumption of 150 W are the radial ones with a slotted configuration (e-h). Among these, even if they allow for the highest torque densities, the Halbach arrangements without rotor yoke have been discarded due to the following disadvantages: higher magnet volume and cost, more complex mechanical integration needed and the potential electromagnetic noise propagated by the stray flux escaping from the outer diameter, as the magnetic field is not confined inside a magnetic yoke, like in the yoked configurations. In order to better compare the two remaining dispositions, namely the radial flux surface-mounted magnet outer and inner rotor configurations, a preliminary mechanical design has been sketched for both topologies, leading to the results in terms of weight and machined parts gathered in Table 3.

**Table 3.** Preliminary mechanical design data for the radial flux surface-mounted magnet configurations.

Configuration	Weight [kg]	# of machined parts
Outer rotor	4.79	5
Inner rotor	3.49	3

From Table 3 it can be extracted that, in terms of weight and number of machined parts, the best option is the inner rotor configuration. However, since the outer rotor choice is more appropriate for an automatic winding process, it was decided to continue with configuration (f), Figure 2 (f), for further investigation.

**Figure 4.**
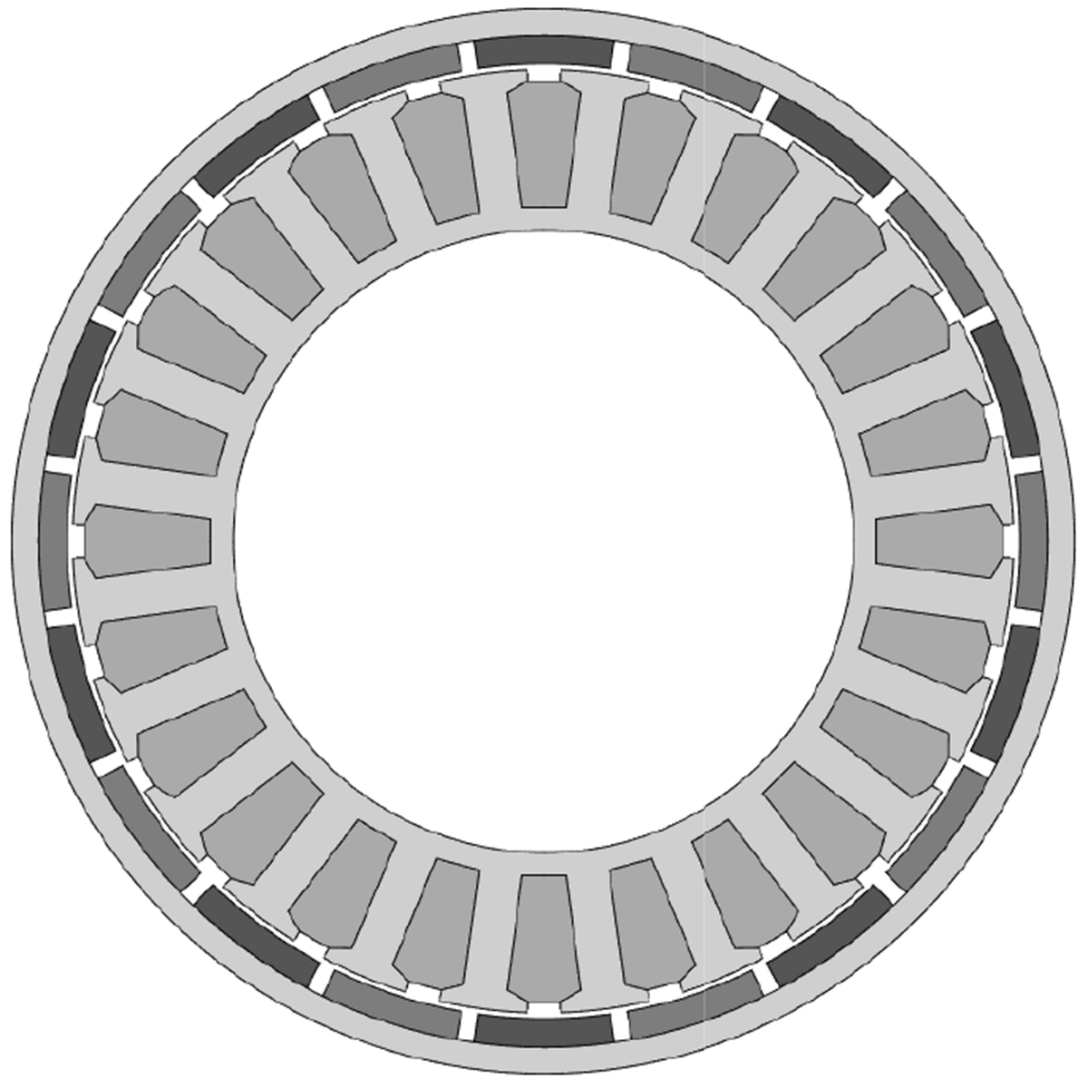
Final electromagnetic design corresponding to 44 mm stack length.

The design candidate which is able to deliver 8 N·m with the least weight has been selected from the Pareto front presented in Figure 3. To preliminary check the thermal behaviour and the modelling hypotheses of the MODE optimization, the stator corresponding to said design has been additively manufactured in 316L stainless steel and the matching winding has been incorporated. A 120 seconds heating test has been performed feeding the machine with the current level computed in the FE simulations. After the completion of the test, it has been noticed that the maximum temperature achieved by the winding is significantly lower than the maximum limit of 120 °C and power consumption is also below the limit established by the requirements. Then, the motor stack length has been reduced from 55 mm to 44 mm to push the motor design closer to the application limits and get the lowest weight possible. The final electromagnetic design is illustrated in Figure 4.

### 2.3 Mechanical design

The demanding maximum weight requirement of the application has made it necessary to carry out a thorough study of the mechanical solution. To this end, this section presents the preliminary mechanical design considered, the results of a Topology Optimization of the structural parts and the final mechanical solution.

**Figure 5.**
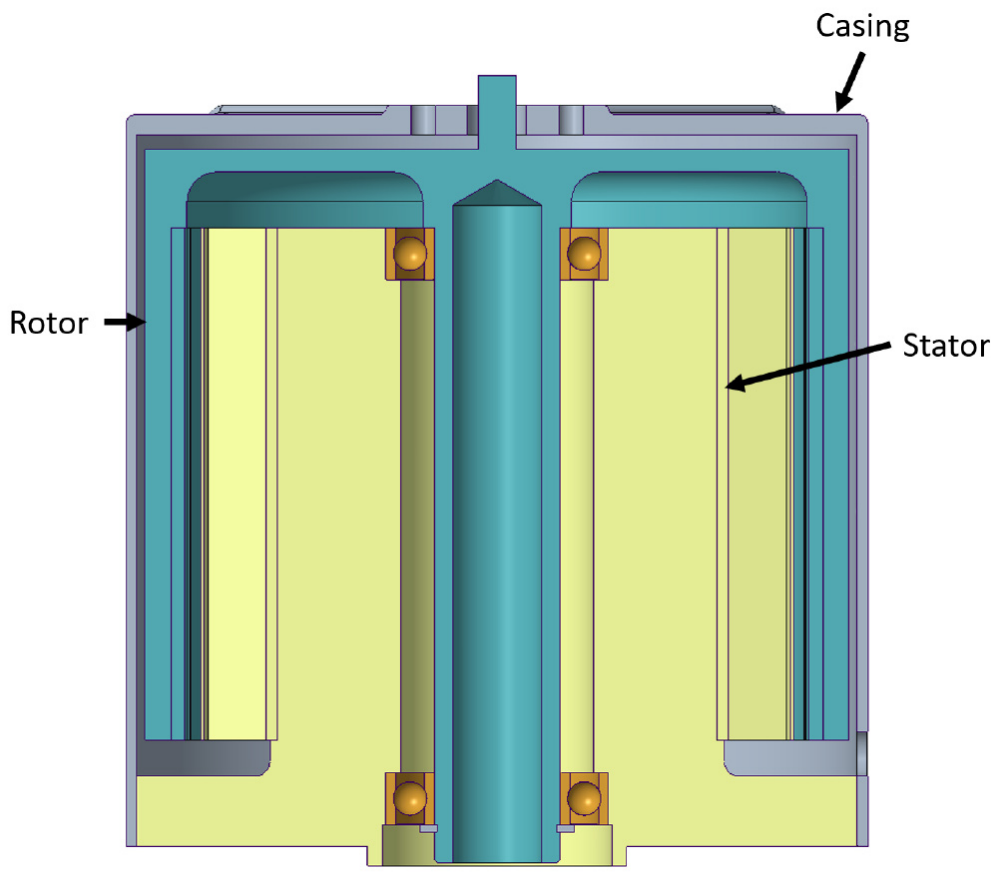
Initial mechanical design layout for the actuator.

***2.3.1 Preliminary mechanical design.*** As stated, a first mechanical design for the actuator has been developed, shown in Figure 5. The assembly has been designed in such a way that the rotor and stator parts have both magnetic and structural functions, with the aim of reducing the weight of the actuator as much as possible, eliminate intermediate parts and joints and maximise reliability. The regions with mechanical function have been oversized so that the later TO has material to remove.

***2.3.2 Topology optimization.*** In order to find the optimum geometry that minimises the actuator weight, while maintaining the mechanical integrity, a mechanical TO algorithm from ANSYS Mechanical ^®^ has been applied to the rotor and stator parts presented in Figure 5. Although different TO approaches for electromagnetic components have been described in the literature ^23– 27^, in this case it has been decided not to apply TO to the active parts of the rotor and the stator as the soft-magnetic regions are already deeply saturated after the parametric optimisation processes described in Section 2.2. The motor casing has also not been topology optimized due to the thin walls and the low magnitude forces it must sustain. The mechanical loads considered in the mechanical TO are as follows:

- **Radial magnetic load:** peak value computed from FEA. Implemented as a uniform radial pressure of 0.56 MPa in the outer surface of the stator and the inner surface of the rotor.
- **Tangential magnetic load:** a uniform tangential pressure corresponding to the rated torque of 8 N·m with a 150% safety factor (total 12 N·m) has been defined for both rotor and stator.
- **Axial load:** an axial load coming from the universal joint, estimated at 280 N has been considered at the input shaft.
- **Weight of the brake and resolver:** the force corresponding to the weights of the brake and resolver, which hang from the motor rotor, estimated as 25 N have been included.
- **Centrifugal force:** for the rotor, the centrifugal force proportional to the square of the angular velocity has been included (87 rad/s as maximum absolute speed of the rotor during operation).

**Figure 6.**
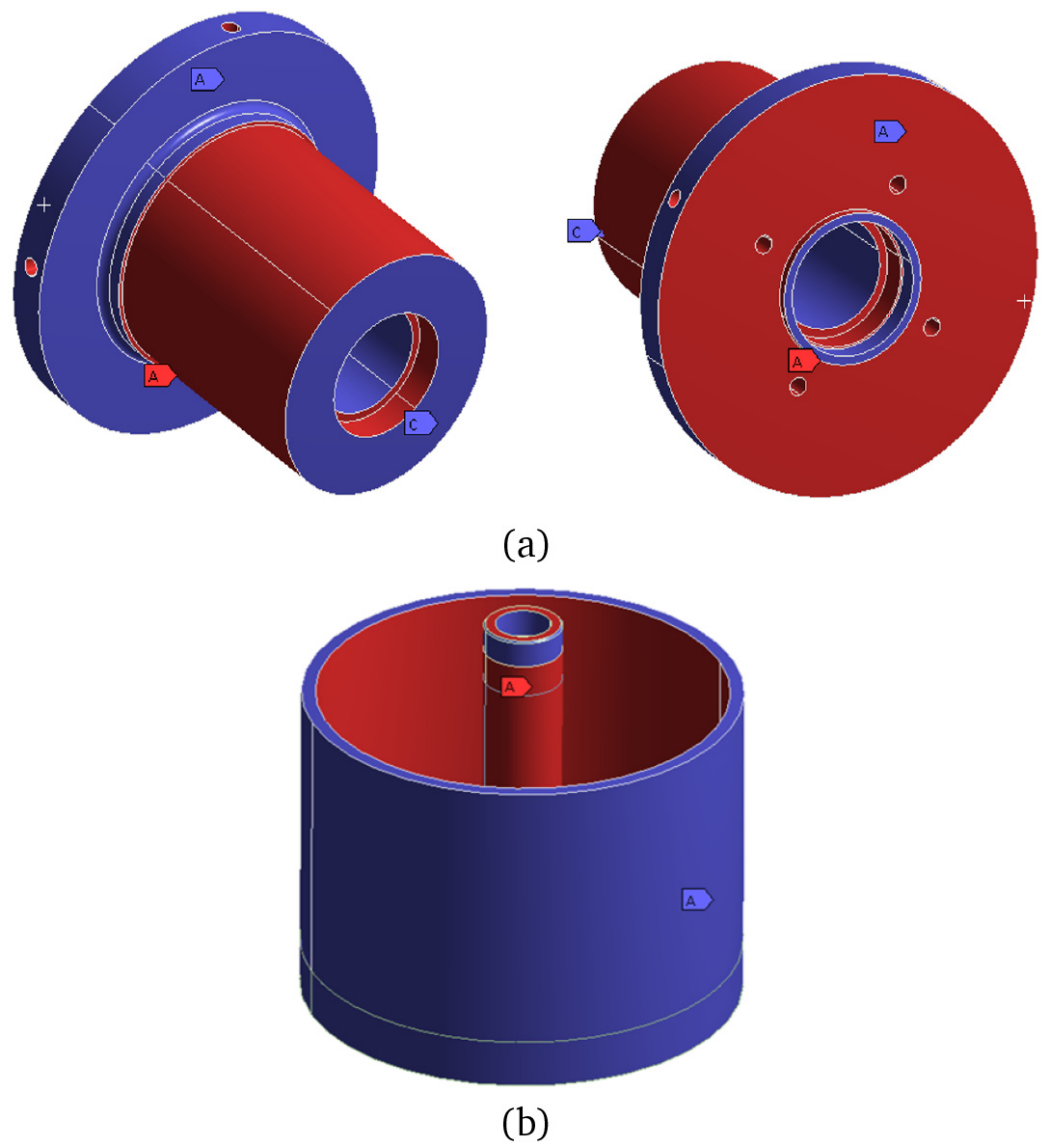
Definition of the frozen surfaces for the mechanical TO (red = frozen, blue = free) ( **a**) stator without its magnetic region ( **b**) rotor and shaft.

For the TO, the electromagnetically active regions (windings, stator teeth and rotor and stator yokes) depicted in Figure 4 have been subtracted to ensure the required electromagnetic performance. Additionally, the interfaces with other actuator components such as the universal joint, the brake and the bearing seats have defined as *frozen*, which means that these surfaces are maintained unchanged along the optimization. A graphical definition of the frozen surfaces of the rotor and stator parts to be topologically optimised is shown in Figure 6.

**Figure 7.**
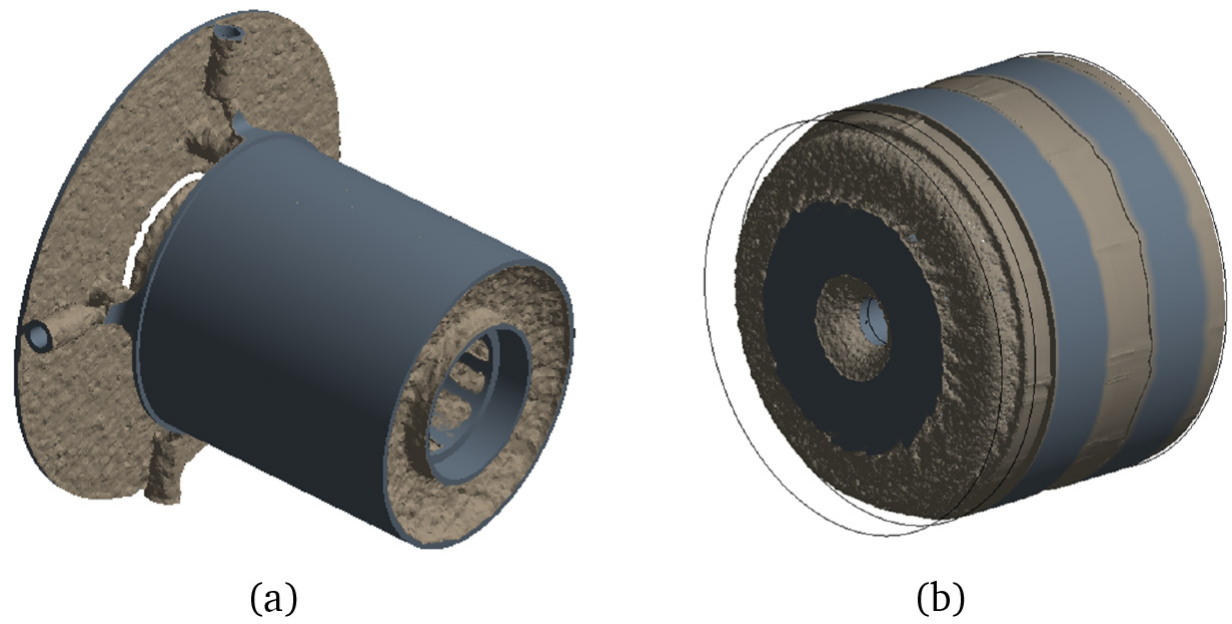
Geometries obtained by the mechanical TO ( **a**) stator ( **b**) rotor and shaft.

After having defined the boundary conditions and acting forces and performing the TO, the geometries obtained are illustrated in Figure 7. In this figure, the newly defined border between material and void is shown in brown, whereas the regions that have been maintained from the initial model are the ones in grey colour. The mass reduction achieved by the TO algorithm is of 80% for the stator and of 42% for the rotor.

By examining the previous figure, it can be concluded that the actuator is mechanically lightly loaded, as the TO algorithm tries to convert almost all the material into void, leaving in some cases ultra thin walls that would be difficult to produce even by additive means. It is then concluded that, for the case study analysed, manufacturability constraints, such as the minimum processing width, are key for defining feasible lightweight structures.

***2.3.3 Manufacturing considerations and constraints.*** To account for the aforementioned constraints, the following modifications have been applied to the final mechanical design.

**Figure 8.**
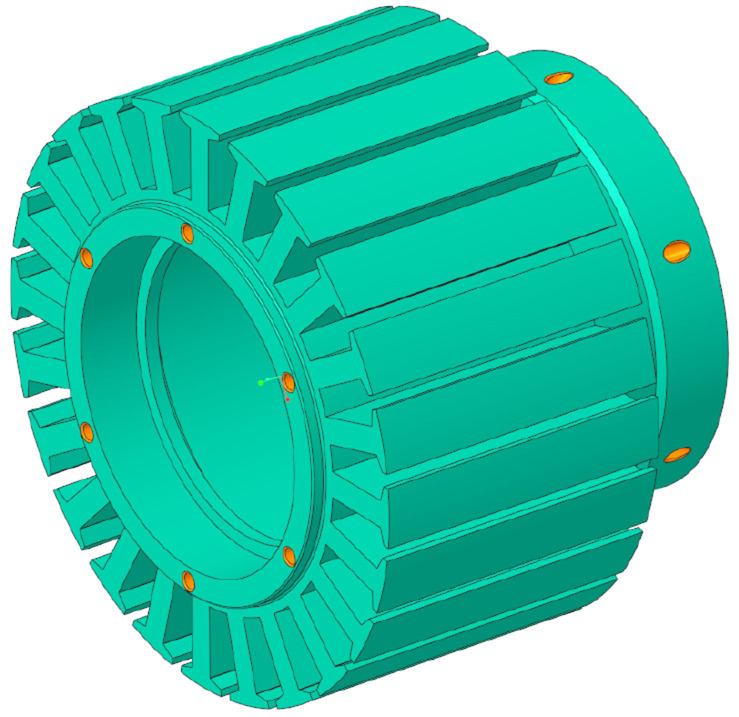
Holes for routing thermal sensor (Pt100) wires.

- First, due to a change in specifications, the casing has been made rectangular to keep the same interface as an existing conventional motor design.
- The previously defined stator part has been split into two parts; namely the stator and a cover. This change has been applied to avoid having a single part with a sudden transition from a massive base to a slender "tower" shape, as the difference in cooling rates among layers when additively manufacturing the part could create excessive internal stresses that caused distortion or cracks in the produced component. The stator has been left to be L-PBF processed in Permendur with magnetic and structural function (housing of the bearings and torque transmission) and it has been decided for the cover to be machined from aluminium to reduce weight.
- The internal diameter of the stator has been defined as the minimum one required to maintain the electromagnetic performance. Two 45º flanges have been incorporated to transition from this diameter to the bearing seats.
- Several curvilinear holes have been added on to the stator to make it possible to route thermal sensor wires and, thus, be able to monitor the temperatures from the least accessible end-winding; see Figure 8. These holes could not have been added in conventional manufacturing.
- The ultra-thin walls between the rotor and the shaft resulting from the TO are hardly manufacturable. To ensure mechanical integrity, while reducing component mass, the union between the magnetic rotor and the torque transmitting shaft has been designed as a spider-web with enough nerves to be manufactured.
- Taking advantage of the complicated shapes attainable by AM, the casing has been designed to incorporate all the required holes for interfacing the several parts and actuator components, while reduce its mass to the maximum. This part would have been almost impossible to produce via non-additive manufacturing methods.

**Figure 9.**
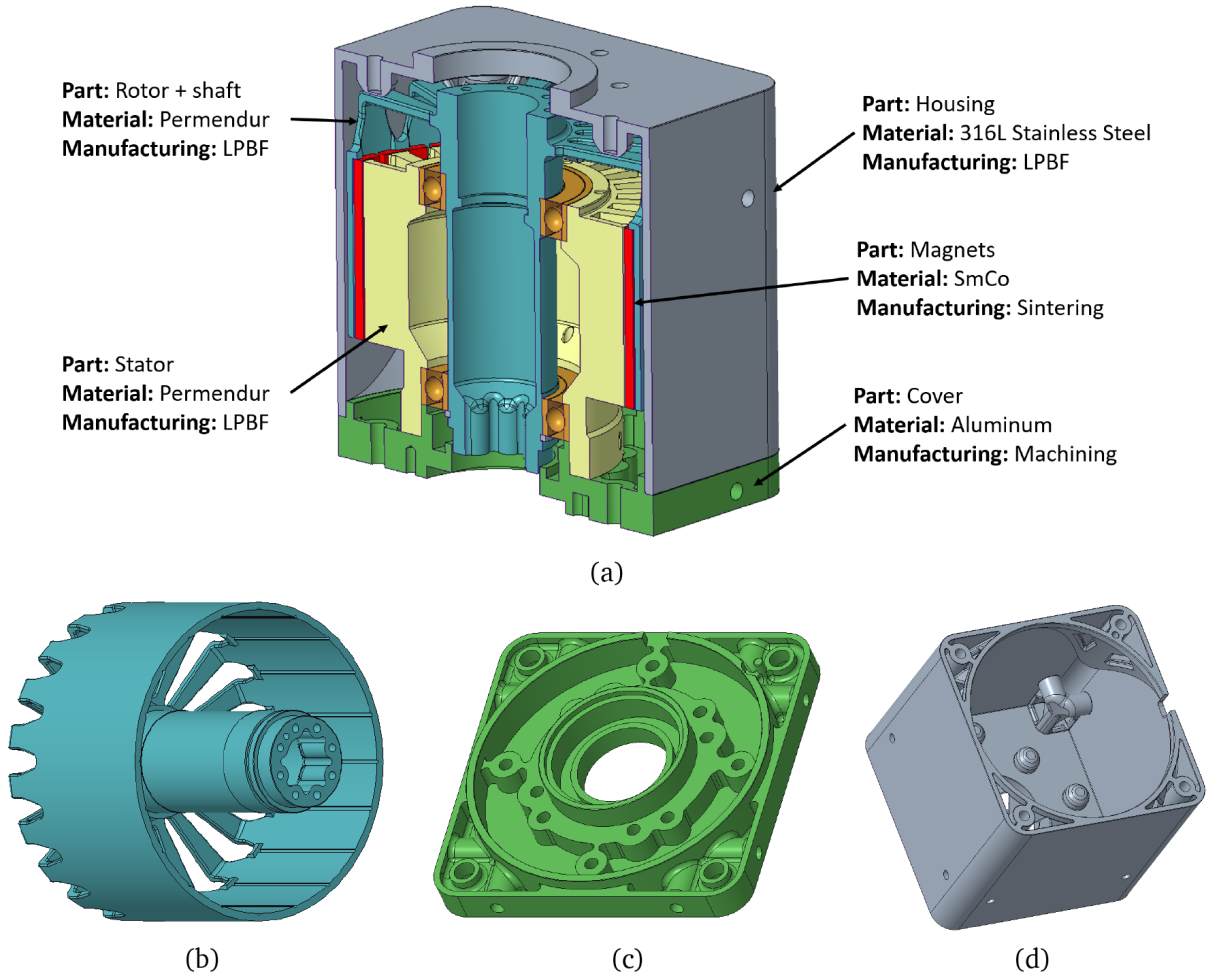
Final mechanical design ( **a**) whole motor section ( **b**) rotor and shaft ( **c**) cover ( **d**) casing.

The final actuator design is presented in Figure 9, in which each part is described together with its corresponding material and manufacturing process.

### 2.4 Prototype manufacturing

**Figure 10.**
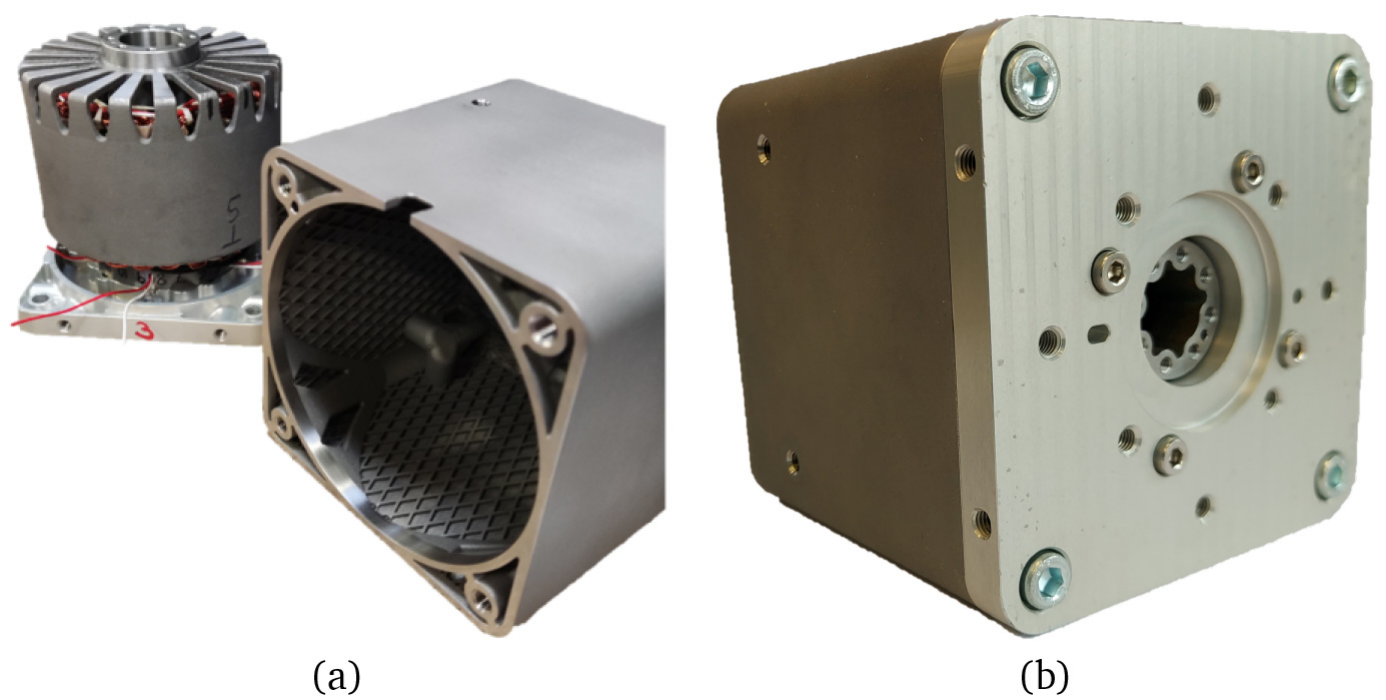
Prototype manufacturing ( **a**) separated components ( **b**) assembled prototype.

Three different actuator prototypes have been manufactured. The AM parts have been produced by Egile Mechanics via LPBF/SLM in a Renishaw AM 400 machine. For the parts manufactured in Permendur, a heat treatment followed by a controlled cooling rate has been applied to balance the desired electromagnetic and mechanical performance ^32^. Afterwards, the components have been separated from the baseplate via Electrical Discharge Machining (EDM). The motor casings have been LPBF processed using gas-atomised 316L stainless steel powder and have been heat treated to relieve stresses according to standard AMS 2759-4. All components have been sandblasted and, after performing the required machining operations, compliance with the required dimensions and tolerances has been checked. The three stators have been wound with enamelled copper wire and impregnated with insulating resin. Sintered SmCo magnets have been glued to the rotors and the covers have been machined from an aluminium block. Finally, the whole electrical machine prototypes have been assembled, as illustrated in Figure 10. Additionally, two of the motor units (prototypes #2 and #3) have been equipped with a DB25 connector in which the power connectors and terminals for PT100 temperature sensors have been integrated. The actuator cable length is approximately 150 mm for prototype #1 and 1000 mm for prototypes #2 and #3.

## 3 Results

**Figure 11.**
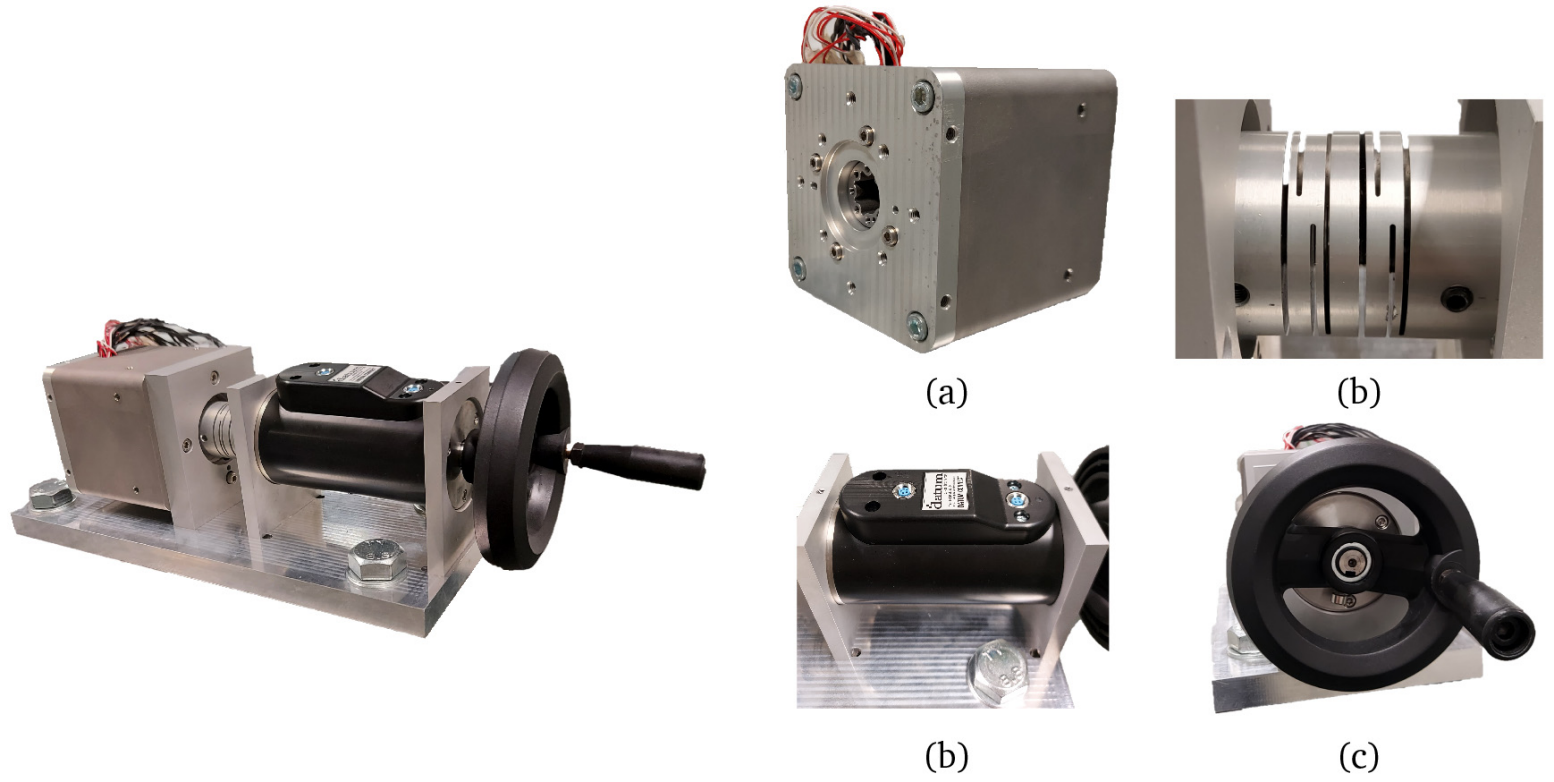
Test bench configuration ( **a**) actuator ( **b**) elastic coupling ( **c**) torquemeter ( **d**) crank.

To validate the electromagnetic and mechanical design, in addition to the manufacturing method, the dedicated test bench from Figure 11 has been installed to test the prototypes. The test setup includes a 20 N·m torquemeter (Datum Electronics M425), an elastic coupling to connect it to the shaft of the actuator and a crank for slowly rotating the actuator manually. The motor phases have been sensed with voltage and current probes and monitored with the aid of a data acquisition equipment (Yokogawa SL1000). The temperature measurements from the Pt100 sensors have been registered with a separate data logger switch unit (Agilent 34972A).

### 3.1 Weight measurements

One of the key goals of this study has been to achieve an actuator as lightweight as possible, with an established goal of *≤* 3 kg. In Table 4, weight measurements conducted for the three prototypes are included.

**Table 4.** Measured mass for the prototypes.

Prototype	#1	#2	#3
**Weight [kg]**	3.10	3.15	3.20

As seen in Table 4, the total weight of all the prototypes is above the goal of 3 kg established in Table 1. Nevertheless, it has to be pointed out hat the torque density of the actuator is very remarkable; significantly lower than that of a pre-existing solution based on conventional manufacturing technologies. Additionally, the measured weight includes the additional cable length and DB25 connectors for prototypes #2 and #3.

### 3.2 Resistance measurements

Phase resistance for the prototypes has been measured at room temperature (about 22 °C) with an RLC-meter (Hioki 3522-50) to check for phase unbalance and comparison against design estimations. The results obtained are collected in Table 5.

**Table 5.** DC phase resistances measured for each prototype.

DC phase resistance [mΩ]	Prototype 1	Prototype 2	Prototype 3
**Phase A**	164.8	196.2	197.8
**Phase B**	165.5	196.1	199.9
**Phase C**	165.3	196.2	198.3
**Phase D**	165.0	198.2	200.1
**Phase E**	164.6	196.3	200.3
**Phase F**	164.7	195.5	203.5

It can be noticed by examining the measurement results that no significant unbalance exists among the different phases and that the addition of the cable over-length and DB25 connector for prototypes #2 and #3 significantly increases the resistance. The phase resistance value estimated during the design stage has been of 185.6 mΩ, not considering the 1000 mm cable over-length. Thus, the values obtained are considered valid and reasonable.

### 3.3 Magnet flux linkage measurements

To estimate the flux linkage produced by the magnets, a standard open-circuit test has been conducted on the three prototypes by spinning the crank and recording the induced voltages. Knowing that the back-EMF is the derivative of the flux linkage with respect to time, the registered values have been post-processed to obtain the flux linkage figures (peak values of the first harmonics for each flux linkage curve). The computed values are gathered in Table 6. The value computed by FEA during the design stage is of 18.95 mWb, which makes the comparison between simulated and experimental values very good.

**Table 6.** Magnet flux linkage at open-circuit conditions.

Magnet flux linkage [mWb]	Prototype 1	Prototype 2	Prototype 3
**Phase A**	18.66	18.76	18.70
**Phase B**	18.62	18.58	18.58
**Phase C**	18.59	18.64	18.41
**Phase D**	18.69	18.76	18.68
**Phase E**	18.64	18.61	18.53
**Phase F**	18.60	18.67	18.52

### 3.4 Quasi-static torque measurements

Standstill torque measurements have been carried out to assess the actuator performance. To do so, the machine has been fed with a DC current corresponding to the rated peak current (14.2 A) in phases A and D and half of the current in the remaining four phases. Afterwards, the crank has been moved as slowly as possible and the current and torque measurements have been registered. As the machine rotates, a maximum torque value is reached, which corresponds to a 90° alignment between the stator and rotor magnetic fields and is essentially the torque capability of the actuator for the tested current level. The results of the test for the three prototypes are recorded in Table 7. The design torque value for the actuator is of 8.00 Nm, meaning that the maximum difference between measured and design values is just of only the 1.38%. This small difference is in concordance with the flux linkage measurement results.

**Table 7.** Quasi-static torque measurements.

Prototype	# 1	# 2	# 3
**Peak torque [N·m]**	8.11	8.00	8.07

### 3.5 Standstill power consumption and temperature measurements

Finally, a DC heating test has been performed with the aforementioned currents on each phase for the three prototypes. The aim of this test has been to check the maximum temperature and DC power consumption after two minutes operating at the maximum current. The motor prototypes have started from a temperature of around 23 °C and the crank has not been operated during the test. The temperatures measured by the Pt100 sensors located in the end-windings of the motor at the end of the test are gathered in Table 8. Additionally, the DC power consumed by each actuator just before switching off the power supply is recorded in Table 9.

**Table 8.** End-winding temperatures after 2 minute DC heating test (malfunctioning Pt100 sensors are indicated by "-").

End-winding temperature [°C]	Prototype 1	Prototype 2	Prototype 3
**Phase A**	84.7	91.3	86.3
**Phase B**	-	59.3	-
**Phase C**	54.8	60.1	58.7
**Phase D**	-	80.0	74.5
**Phase E**	-	58.0	51.1
**Phase F**	-	60.3	54.5

**Table 9.** DC power consumption after 2 minutes.

Prototype	#1	#2	#3
**DC power [W]**	121.0	147.5	148.7

First, in Table 8, it is shown that the temperatures achieved after the 2 minute heating test are far from the allowable temperature limit of 120 °C. The actuator benefits from the reduced number of joints between the stator core and the output cover, as the solid single-component stator provides for an effective thermal dissipation from the coils to the aluminium cover. Regarding DC power consumption, the measured values are very close to the maximum limit of 150 W defined in Table 1, without exceeding it. The power consumed by each prototype is perfectly in line with the measured phase resistance values.

As a conclusion of the prototype testing campaign, the design and manufacture of the actuators is considered a success, as the main design specifications are met.

## 4 Discussion

In this paper, the design, both electromagnetic and mechanical, the manufacturing and the testing of an aerospace actuator manufactured by Laser Powder Bed Fusion (LPBF) is presented. For the electromagnetic design, 8 different machine topologies are analyzed and parametrically optimized and the best alternative in terms of electromagnetic performance, weight and ease of manufacturing is selected. The mechanical design of the actuator is also detailed, starting by an initial mechanical design and refining it via topology optimization. Finally, three actuator prototypes are additively manufactured and tested, showing good agreement between design and test results and an excellent repeatability of the magnetic behaviour of the processed soft-magnetic parts.

The additive manufacturing of motor components has allowed to process parts with dual magnetic and structural function, eliminating the need for additional parts and joining operations, improving thus thermal dissipation and allowing a significant reduction in weight (more than 20 %) compared to an existing conventional solution. Topological optimisation has also proved useful for designing highly lightweight structures. Finally, additive manufacturing has made it possible to add features, such as channels through which to route cable sensors or lattices in the casing, that would have been impossible to manufacture using conventional methods.

In light of the literature review and the work presented in this article, even if additive manufacturing of electrical machines is still in its early phases of development, it shows promising perspectives in terms of repeatability in material properties, ability to process complex 3D multifunctional parts (electromagnetic, thermal and structural), having lightweight topology-optimized structural parts for increased power density and material savings, and allowing an increased performance of cooling systems via a higher degree of integration and feature inclusion. Additional potential benefits to be further analyzed include the possibility of having tuned material properties (e.g. anisotropic electrical and thermal conductivities), free-form coils with high fill factors, reduced end-windings and AC losses, and the direct deposition of high temperature insulation materials.

## 5 Conclusions

The study carried out in this research, together with the results obtained, highlight the great potential of additive manufacturing for the production of functional electrical machine components. In the case analysed, parts have been produced that are difficult to manufacture using traditional production methods and that are able to reduce the overall weight of the actuator. Not only the geometric freedom offered by AM, but also the possibility of producing dual-function parts, electromagnetic and mechanical, has been a great advantage in reducing weight and increasing reliability by reducing the number of assembly parts.

## Ethics and consent

Ethical approval and consent were not required.

## Data Availability

No data are associated with this study.
