# Supplementary material for: Design and additive manufacturing of a lightweight aerospace electric actuator
Source: Open Res Eur. 2025 Feb 25;4:121. Originally published 2024 Jun 21. [Version 2] doi: 10.12688/openreseurope.17752.2 (PMC11897690; doi:10.12688/openreseurope.17752.2)
Supplement: Peer review history

# Reviewer report: Reviewer response for version 2

**DOI:** 10.21956/openreseurope.20104.r51924

**Published:** 2025-03-13

**Authors:** Uğur Demir

**Affiliations:** 1 Marmara University, Istanbul, Turkey

Dear Author,

 The comment below #1 was not answered reasonably. The required information should be added at 3rd round.

 #1: “the results of the study should improve with a comparison of a shelf product which is similar according to the requirements”

 The comment below #2 was answered partially. Because the stated references  should be discussed to improve the quality of your manuscript at 3rd round.

 #2 : “ the design optimization techniques for the actuator should be discussed”

 The comment below #3 was not answered. Because the optimization algorithm was not explained in detail. What are objective, design constraints, goal, and etc?  It should be added at 3rd round.

 #3 : Furthermore, the design optimization techniques for the actuator should be discussed.

 Besides, topology optimization is dealt with the design of experiments in terms of the parametric design and objectives.

 References

 1- Demir U, 2021 (Ref 1)

***This reference provides an aspects for your project because this paper shows a methodology for lightweight of an actuator/electric machines.***

 2- Kocabicak Z, et al., 2020 (Ref 2)

***This reference provides an aspects for your project because this paper  not only shows a methodology for lightweight of an actuator/electric machines and also design of experiments.***

 3- Demir U, et al., 2021 (Ref 3)

***This reference provides an aspects for your project because this paper  consider also additive materials such as SOFT MAGNETIC COMPOSITE for actuators.***

 4- DEMİRCİ B, et al., 2023 (Ref 4)

***This reference provides an aspects for your project because this paper not only shows a methodology for lightweight of an actuator/electric machines and also topology optimization.***

 5- Demir U, et al., 2023 (Ref 5)

***This reference provides an aspects for your project because this paper presents the detailed multi objective optimization for the actuators.***

 6- Demir U, et al., 2024 (Ref 6)

***This reference provides an aspects for your project because this paper provides system based optimization for the actuators.***

 Best

Is the study design appropriate and does the work have academic merit?

Yes

Is the work clearly and accurately presented and does it cite the current literature?

Yes

If applicable, is the statistical analysis and its interpretation appropriate?

Not applicable

Are all the source data underlying the results available to ensure full reproducibility?

Yes

Are the conclusions drawn adequately supported by the results?

Partly

Are sufficient details of methods and analysis provided to allow replication by others?

Yes

Reviewer Expertise:

UĞUR DEMİR received the Ph.D. degree in mechatronics engineering from Marmara University, in 2018. He is currently an Associate Professor with the Department of Mechatronics Engineering, Marmara University. He has extensively worked on the development of novel electrical machine technologies and the actuator for electric vehicle traction operations and mechatronics systems. His research interests include the electromagnetic design of electrical machines, design optimization, artificial neural network applications, autonomous driving, and advanced driving technologies.

I confirm that I have read this submission and believe that I have an appropriate level of expertise to confirm that it is of an acceptable scientific standard, however I have significant reservations, as outlined above.

## References

[1] : Improvement of the power to weight ratio for an induction traction motor using design of experiment on neural network. *Electrical Engineering* .2021;103(5) : 10.1007/s00202-020-01204-2 2267-2284 10.1007/s00202-020-01204-2

[2] : Design and optimization of an electromechanical actuator for the latch of a foldable vehicle seat. *Materials Testing* .2020;62(7) : 10.3139/120.111539 749-755 10.3139/120.111539

[3] : Performance assessments of the material for the traction motor cores of an electric racing kart. *Materials Testing* .2021;63(6) : 10.1515/mt-2020-0085 519-528 10.1515/mt-2020-0085

[4] : Multi System Level Driving Scenarious Based Topology Optimization of Bracket Design for 2 DoF Vehicle Simulator. *International Journal of Automotive Science and Technology* .2023;7(3) : 10.30939/ijastech..1244113 248-258 10.30939/ijastech..1244113

[5] : Comparative Design Improvement of the Growing Rod for the Scoliosis Treatment Considering the Mechanical Complications. *IEEE Access* .2023;11: 10.1109/ACCESS.2023.3268147 40107-40120 10.1109/ACCESS.2023.3268147

[6] : Intelligent Design Optimization for Traction and Steering Motors of an Autonomous Electric Shuttle under Driving Scenarios. *Electronics* .2024;13(3) : 10.3390/electronics13030566 10.3390/electronics13030566

# Reviewer report: Reviewer response for version 2

**DOI:** 10.21956/openreseurope.20104.r51576

**Published:** 2025-03-11

**Authors:** Adrien Thabuis

**Affiliations:** 1 Ecole Polytechnique Federale Lausanne (EPFL), Lausanne, Switzerland

I have no further comments to make.

Is the study design appropriate and does the work have academic merit?

Yes

Is the work clearly and accurately presented and does it cite the current literature?

Yes

If applicable, is the statistical analysis and its interpretation appropriate?

Not applicable

Are all the source data underlying the results available to ensure full reproducibility?

No

Are the conclusions drawn adequately supported by the results?

Partly

Are sufficient details of methods and analysis provided to allow replication by others?

No

Reviewer Expertise:

Elecromagnetic devices, Additive manufacturing, topology optimization

I confirm that I have read this submission and believe that I have an appropriate level of expertise to confirm that it is of an acceptable scientific standard.

# Reviewer report: Reviewer response for version 1

**DOI:** 10.21956/openreseurope.19192.r43296

**Published:** 2024-09-10

**Authors:** Adrien Thabuis

**Affiliations:** 1 Ecole Polytechnique Federale Lausanne (EPFL), Lausanne, Switzerland

The article covers the design steps of an electromagnetic (EM) actuator. Multiple topologies are considered and optimized independently for better torque and weight. A machine is selected based on different criteria. Then, focus is put on the mechanical integrity of the device and various methods are explored to reduce its mass.

 Next are a few comments I suggest you address for full approval.

1. In the introduction, I would suggest explaining better why reducing the weight/mass of pieces in the aeronautical sector helps mitigate fuel consumption. (I know why but), I think this would allow the reader to focus on that aspect from the start.
2. Please be more careful when using the term weight and mass. We are scientists, they have different meanings. I would suggest using mass everywhere.
3. Better discuss the choice of material, even if a reference is provided, we need to know why you chose this material. A LOT of mass could be saved by considering another material, which could remove the need for such an analysis (or not, but we need to know if you considered it).
4. In the introduction, you criticize the lack of a full system entirely 3D-printed but still uses conventional PM and conductors. There are some works in the literature already printing the iron parts, so there are no real improvements regarding this aspect. Please rephrase.
5. “ All the depicted shapes have been optimized parametrically in terms of FEA,” → this does mean anything. Be more precise or remove this sentence.
6. Not clear which physics are solved by the FEA. Magnetostatics or other?
7. The material types used in the simulation are provided. It would be nice to also provide some actual values like remanence of PM and saturating field for the iron alloys
8. When stating “In order to better compare the two remaining dispositions, namely the radial flux surface-mounted magnet outer and inner rotor configurations, a preliminary mechanical design has been sketched for both topologies, leading to the results in terms
  of weight and machined parts gathered in Table 3.” → please add the corresponding letters in from Figure 2 and 3 for clarity.
9. It seems that many of the topologies could have been discarded prior to the analysis. The ease of automatic winding and issues coming from the Halbach arrangement could have been known prior to that → why still considering them?
10. More details are needed for the thermal “check”. We don’t know how this has been performed, how was the temperature monitored, was it a realistic case? Was it performed with everything assembled or just the stator by itself? Which “cooling conditions”, natural convection? → if so we need to know the environment or integration. This is even more critical as the considered topology is an outer rotor configuration which can be more demanding it terms of thermal management.
11. In Figure 4, it would be nice to add some sizes to better grasp the footprint of the actuator.
  → this helps estimate if manufacturing is realistic.
  A view of the magnetic flux density distribution would also help estimate if the EM design is properly done (saturated regions).
12. In the topology optimization part 2.3.2, it is not clear if there has been a constraint on the part stiffness or not? How do you ensure not having the trivial solution of a completely empty part? → what are the constraints to the optimization problem?
13. Figure 7 does not present much information, some “cut-view” could better help visualizing the newly generated design.
14. Most of the modifications proposed in 2.3.3 part could have been integrated within the TO if explored more deeply. Changing so many things after the optimization makes me question the usefulness of such an approach. Considering such constraints beforehand could have maybe resulted in a very different topology.
15. It would be nice to know which insulating resin has been used. This would affect the thermal properties too.
16. How is this motor driven? Are there some Hall sensors?
17. Page 11, there is a typo just before the 3.2 part. “it has to be pointed out hat the torque […]”, it should be “it has to be pointed out **t**hat the torque”. Please pass over the whole manuscript to avoid such mistakes. There are many free tools that can help you with that …
18. It is said that the pre-existing solution was heavier but precised how much. It would help better appreciate the improvement.
19. Why are the phase resistances of Proto1 lower than the others?
20. In the conclusion: “Not only the geometric freedom offered by AM, but also the possibility of producing dual-function parts, electromagnetic and mechanical, ”.
  Maybe I missed a part but this does not like being the case to me. It seems that you removed all of the parts strongly contributing to the EM performances from the mechanical analysis.
21. Great work overall, congratulations

Is the study design appropriate and does the work have academic merit?

Yes

Is the work clearly and accurately presented and does it cite the current literature?

Yes

If applicable, is the statistical analysis and its interpretation appropriate?

Not applicable

Are all the source data underlying the results available to ensure full reproducibility?

No

Are the conclusions drawn adequately supported by the results?

Partly

Are sufficient details of methods and analysis provided to allow replication by others?

No

Reviewer Expertise:

Elecromagnetic devices, Additive manufacturing, topology optimization

I confirm that I have read this submission and believe that I have an appropriate level of expertise to confirm that it is of an acceptable scientific standard, however I have significant reservations, as outlined above.

# Response

**Published:** 2025-02-12

**Authors:** Borja Lizarribar Carrillo

**Affiliations:** Transport and Energy Division, CEIT, San Sebastián, Basque Country, Spain

We provide below a point by point response to reviewer comments:

1. **In the introduction, I would suggest explaining better why reducing the weight/mass of pieces in the aeronautical sector helps mitigate fuel consumption. (I know why but), I think this would allow the reader to focus on that aspect from the start.**

**Response: **Thank you very much for your comment. An explanation of this fact has been introduced in the introduction.

1. **Please be more careful when using the term weight and mass. We are scientists, they have different meanings. I would suggest using mass everywhere.**

**Response: **Thank you very much, weight has been changed to mass in the article.

1. **Better discuss the choice of material, even if a reference is provided, we need to know why you chose this material. A LOT of mass could be saved by considering another material, which could remove the need for such an analysis (or not, but we need to know if you considered it).**

**Response: **In order to explain the material selection, the paper has been changed and the following has been added: “The material selected for these components is Permendur, an iron-cobalt-vanadium alloy. Iron-cobalt alloys are known for their high magnetic saturation, which gives significant advantages in terms of torque density for electrical machines. Furthermore, testers have been manufactured of FeCo and FeSi by Laser Powder Bed Fusion and it has been concluded that FeCo is a better option in the present case in terms of the density achieved and the cracks that occurred in the manufacture of FeSi6.5 alloys **32**. Details regarding material assessment and optimization of process parameters, including thermal treatment, can be found in **32**.”

1. **In the introduction, you criticize the lack of a full system entirely 3D-printed but still uses conventional PM and conductors. There are some works in the literature already printing the iron parts, so there are no real improvements regarding this aspect. Please rephrase.**

**Response: **It is true that work can be found in literature in which iron parts are manufactured via Additive Manufacturing. What we wanted to highlight in the introduction is that there is lack of papers in which the design has been carried out considering that the iron parts of the motor will be manufactured by Additive Manufacturing means. Which has permitted more freedom in the study of motor topologies for the actuator and the design of the active parts. The papers that are found in literature are normally more focused on the materials side and do not explore extensively the advantages that AM brings in terms of geometrical freedom for electrical machines, which has been the main focus of this article. In order to clarify this aspect, the introduction has been modified.

1. **All the depicted shapes have been optimized parametrically in terms of FEA,” → this does mean anything. Be more precise or remove this sentence.**

**​​​​​​​Response: **Thanks for your comment, changes have been made in the article in order to be more precise.

1. **Not clear which physics are solved by the FEA. Magnetostatics or other?**

**​​​​​​​Response: **It is mentioned in the paper: “magnetostatic 2D FE simulations in the software FEMM”.

1. **The material types used in the simulation are provided. It would be nice to also provide some actual values like remanence of PM and saturating field for the iron alloys.**

**​​​​​​​Response: **Permanent magnet remanence was not actually measured in the project, but the permendur polarisation saturation was, J _s_ = 2.26 T. The remanence specified by the supplier of the PM was 1.08-1.10 T. In case it is useful for you I attach the B-h curve measured in the permendur material.  

 B-h curve figure attachment link: https://s3-eu-west-1.amazonaws.com/openreseurope/linked/246983.17752-_B-h_Curve_figure-_Comment_response.png

 ​​​​​​​In any case, to have more information available for the reader, properties of the materials have been added in Table 2.

1. **When stating “In order to better compare the two remaining dispositions, namely the radial flux surface-mounted magnet outer and inner rotor configurations, a preliminary mechanical design has been sketched for both topologies, leading to the results in terms of weight and machined parts gathered in Table 3.” → please add the corresponding letters in from Figure 2 and 3 for clarity.**

**​​​​​​​Response: **Done, thanks.

1. **It seems that many of the topologies could have been discarded prior to the analysis. The ease of automatic winding and issues coming from the Halbach arrangement could have been known prior to that → why still considering them?**

**​​​​​​​Response: **Very true, but the Topic Manager concerns on automatic winding and Halbach configuration came after the assessment of the electrical machine topologies has been done in the project.

1. **More details are needed for the thermal “check”. We don’t know how this has been performed, how was the temperature monitored, was it a realistic case? Was it performed with everything assembled or just the stator by itself? Which “cooling conditions”, natural convection? → if so we need to know the environment or integration. This is even more critical as the considered topology is an outer rotor configuration which can be more demanding it terms of thermal management.**

**​​​​​​​Response: **More details have been given in the article about this topic. The check has been done to a stator “dummy” and it is true that the conditions when it is mounted will be more demanding. However, we considered that due to the maximum temperature achieved, 90 ºC starting from 52 ºC, far from the maximum of 120 ºC, and the lower power consumption, 113 W, we could decrease the effective length so as to reduce the weight of the motor.

1. **In Figure 4, it would be nice to add some sizes to better grasp the footprint of the actuator → this helps estimate if manufacturing is realistic. A view of the magnetic flux density distribution would also help estimate if the EM design is properly done (saturated regions).**

**​​​​​​​Response: **Both things have been added in Figure 4.

1. **In the topology optimization part 2.3.2, it is not clear if there has been a constraint on the part stiffness or not? How do you ensure not having the trivial solution of a completely empty part? → what are the constraints to the optimization problem?**

**​​​​​​​Response: **There has not been a constraint on the part stiffness. To ensure not having the trivial solution you mention we have defined some parts as frozen, so they cannot be removed in the optimisation. These frozen parts are shown in the Figure 6, and they will require at least one mesh element around these zones. No additional constraints were considered. However, this assumption has led to very thin walls at this part that afterwards have been widened as it explained in the article.

1. **Figure 7 does not present much information, some “cut-view” could better help visualizing the newly generated design.**

**​​​​​​​Response: **Thanks for your comment, a cut-view of the stator TO has been added in Figure 7.

1. **Most of the modifications proposed in 2.3.3 part could have been integrated within the TO if explored more deeply. Changing so many things after the optimization makes me question the usefulness of such an approach. Considering such constraints beforehand could have maybe resulted in a very different topology.**

**​​​​​​​Response: **Perhaps the article tries to show the project's chronology the way they were, but it is true a better approach could have been taken in the mechanical TO.

1. **It would be nice to know which insulating resin has been used. This would affect the thermal properties too.**

**​​​​​​​Response: **Ther resin used was epoxy class H.

1. **How is this motor driven? Are there some Hall sensors?**

**​​​​​​​Response: **The actuator incorporates a brake and a resolver in the non-drive end as illustrated in Figure 1.

1. **Page 11, there is a typo just before the 3.2 part. “it has to be pointed out hat the torque […]”, it should be “it has to be pointed out that the torque”. Please pass over the whole manuscript to avoid such mistakes. There are many free tools that can help you with that …**

**​​​​​​​Response: **Thanks, the typo has been corrected and the whole paper cross-checked for mistakes.

1. **It is said that the pre-existing solution was heavier but precised how much. It would help better appreciate the improvement.**

**​​​​​​​Response: **We are sorry but we are not able to share this information due to confidentiality imposed by the topic manager.

1. **In the conclusion: “Not only the geometric freedom offered by AM, but also the possibility of producing dual-function parts, electromagnetic and mechanical, ”. Maybe I missed a part but this does not like being the case to me. It seems that you removed all of the parts strongly contributing to the EM performances from the mechanical analysis.**

**​​​​​​​Response: **That was not exactly what we meant. What we meant was that a manufacturing process (L-PBF) has been used to produce parts that integrate electromagnetic parts (rotor and stator) with structural parts capable of transmitting torque and providing mechanical stability. In a traditional manufacturing process, the soft magnetic parts are usually produced by lamination and additional mechanical parts are required to transmit the torque and ensure mechanical stability. With AM, we have been able to integrate these two parts, reducing the number of parts to be manufactured and the number of joints between parts.

# Reviewer report: Reviewer response for version 1

**DOI:** 10.21956/openreseurope.19192.r42571

**Published:** 2024-09-05

**Authors:** Chi Ma

**Affiliations:** 1 Chongqing University, Chongqing, Chongqing, China

**Review Comments**

**Overall Evaluation:** This paper presents a detailed study on the design and additive manufacturing of a lightweight aerospace electric actuator. The topic is innovative, particularly in the integration of topology optimization and additive manufacturing in lightweight design, showcasing cutting-edge technologies in this field. However, there are areas in certain key technical details where the paper could be further improved.

**Strengths:**

1. **Innovative Design Approach:** The paper demonstrates an innovative approach by combining topology optimization with additive manufacturing, showing significant potential in lightweight design and its application in electric actuators.
2. **Detailed Experimental Validation:** The paper provides comprehensive experimental results that validate the feasibility and effectiveness of the design approach, especially in balancing weight reduction and structural integrity.

**Technical Comments:**

1. **Integration of Performance Optimization with Weight Reduction:**
  - The paper primarily focuses on the goal of weight reduction through topology optimization, but it lacks detailed discussion on how to simultaneously optimize motor performance (e.g., output torque, efficiency, thermal management). While weight reduction can enhance the lightweight characteristics of the motor, it may lead to performance degradation or instability if electromagnetic performance is not adequately considered.
  - It is recommended that the authors more closely integrate motor performance optimization with the goal of weight reduction during the topology optimization process. For instance, a multi-objective optimization approach could be employed, where performance indicators (such as torque density, losses, and temperature rise) are included as constraints or objective functions alongside structural weight. This would ensure that the design achieves weight reduction without significantly compromising motor performance.
2. **Details on Electromagnetic Design:**
  - Although the paper compares different motor topologies, it provides limited discussion on the detailed behavior of each topology under various operating conditions (e.g., different speeds or loads). The authors are encouraged to further explore the differences in electromagnetic characteristics under these conditions, particularly in terms of induced electromotive force, loss characteristics, and efficiency.
3. **Thermal Management and Heat Dissipation Analysis:**
  - It is suggested to supplement or expand the discussion on heat flux density, thermal resistance network models, and potential localized overheating phenomena in actual operation to ensure that the optimized design maintains reliability under extreme conditions.
4. **Limitations of Topology Optimization:**
  - While topology optimization effectively reduces component weight, the paper mentions that some ultra-thin structures may be difficult to manufacture. It is recommended to further analyze the dynamic performance of the optimized structure during actual operation (such as vibration response and modal analysis) and explore potential improvement measures.

**Conclusion and Recommendations:** Overall, this paper provides valuable insights into the design and manufacturing of aerospace electric actuators. However, there is room for improvement in the integration of design methods, particularly in combining performance optimization with weight reduction. The authors are encouraged to conduct major revisison based on the above technical comments to further enhance the research content.

Is the study design appropriate and does the work have academic merit?

Partly

Is the work clearly and accurately presented and does it cite the current literature?

Partly

If applicable, is the statistical analysis and its interpretation appropriate?

Partly

Are all the source data underlying the results available to ensure full reproducibility?

Partly

Are the conclusions drawn adequately supported by the results?

Partly

Are sufficient details of methods and analysis provided to allow replication by others?

Partly

Reviewer Expertise:

Topology optimization, intelligent design and optimization

I confirm that I have read this submission and believe that I have an appropriate level of expertise to confirm that it is of an acceptable scientific standard, however I have significant reservations, as outlined above.

# Response

**Published:** 2025-02-12

**Authors:** Borja Lizarribar Carrillo

**Affiliations:** Transport and Energy Division, CEIT, San Sebastián, Basque Country, Spain

e provide below a point by point response to reviewer comments::

1. **The paper primarily focuses on the goal of weight reduction through topology optimization, but it lacks detailed discussion on how to simultaneously optimize motor performance (e.g., output torque, efficiency, thermal management). While weight reduction can enhance the lightweight characteristics of the motor, it may lead to performance degradation or instability if electromagnetic performance is not adequately considered.**

Thank you for your comments. It is true that the optimisation of the electrical machines does not take into account simultaneously the different physics affecting the machine performance, thermal, electromagnetic and mechanical. A further analysis of the topologies studied would require the simultaneous optimisation of the electromagnetic-thermal-mechanical physics. In any case, the objective of the European project and the article was not to find a global optimum solution, but to explore the benefits that additive manufacturing can bring to the design of actuators.

1. **It is recommended that the authors more closely integrate motor performance optimization with the goal of weight reduction during the topology optimization process. For instance, a multi-objective optimization approach could be employed, where performance indicators (such as torque density, losses, and temperature rise) are included as constraints or objective functions alongside structural weight. This would ensure that the design achieves weight reduction without significantly compromising motor performance.**

As it has been said for the previous comment, we agree that integrating different physics in the topology optimisation would have been the best option to achieve a globally optimised design without compromising motor performance. However, this was not the objective of the project or the paper.

1. **Although the paper compares different motor topologies, it provides limited discussion on the detailed behavior of each topology under various operating conditions (e.g., different speeds or loads). The authors are encouraged to further explore the differences in electromagnetic characteristics under these conditions, particularly in terms of induced electromotive force, loss characteristics, and efficiency.**

Thank you for your comment. The requirements of the project were only those relating to nominal torque, maximum DC consumption and weight. There were no requirements for different speeds or loads. Moreover, the operation of this motor is quasi-static and the losses are mainly those generated in the stator copper, with the exception of the losses associated with the ripple of the current converter, for which no data were available, but with an estimated switching frequency of over 40 kHz. In this case, the study of the different configurations for partial loads is not very interesting, while the losses vary in proportion to the square of the current. In case you find it interesting, we attach here the back EMF estimated by the FEA simulation of the 4 cases that reach the requirement of 8 Nm. The speed considered for these models is 400º/s and the specific candidates shown for each configuration are those that achieve 8 Nm with the defined maximum DC power consumption. It is interesting to note from this graph that while all the electromagnetic parameters have not been considered in the optimisation, high amplitude harmonics can be seen in the back EMF waveform of the external rotor configuration. 

https://s3-eu-west-1.amazonaws.com/openreseurope/linked/246467.17752-Author_response_to_Reviewer__Chi_Ma_v1.pdf

1. **It is suggested to supplement or expand the discussion on heat flux density, thermal resistance network models, and potential localized overheating phenomena in actual operation to ensure that the optimized design maintains reliability under extreme conditions.**

Thank you for your comment. We have added a thermal analysis from MotorCAD® before section 2.3 showing the average and hotspot of the copper winding. Two figures, Figures 12 and 13, of the thermal model used in the study have been included.

1. **While topology optimization effectively reduces component weight, the paper mentions that some ultra-thin structures may be difficult to manufacture. It is recommended to further analyze the dynamic performance of the optimized structure during actual operation (such as vibration response and modal analysis) and explore potential improvement measures.**

While the operation of the electric machine design is quasi-static, not dynamic, there were not requirements in terms of vibration response, thus no structural dynamic analysis has been done.

1. **Overall, this paper provides valuable insights into the design and manufacturing of aerospace electric actuators. However, there is room for improvement in the integration of design methods, particularly in combining performance optimization with weight reduction. The authors are encouraged to conduct major revisison based on the above technical comments to further enhance the research content.**

Thank you very much, we have tried to respond all your concerns.

# Reviewer report: Reviewer response for version 1

**DOI:** 10.21956/openreseurope.19192.r43295

**Published:** 2024-08-30

**Authors:** Uğur Demir

**Affiliations:** 1 Marmara University, Istanbul, Turkey

Dear Author,

 I think the manuscript is acceptable because the manuscript is comprehensive manner and it provides novelty. Therefore it contributes to literature. However, the results of the study should improve with a comparison of a shelf product which is similar according to the requirements. Furthermore, the design optimization techniques for the actuator should be discussed. Besides, topology optimization is dealt with the design of experiments in terms of the parametric design and objectives.

 This paper presents design optimization of additive manufacturing-based actuator considering the lightweight requirements for aerospace. In terms of the design requirements, the topology optimization for the actuator is dealt with. Besides, 3 different prototypes are performed. The study is completed with comparative analysis. In conclusion, the paper presents 20% reduction for lightweight.

Is the study design appropriate and does the work have academic merit?

Yes

Is the work clearly and accurately presented and does it cite the current literature?

Yes

If applicable, is the statistical analysis and its interpretation appropriate?

Not applicable

Are all the source data underlying the results available to ensure full reproducibility?

Yes

Are the conclusions drawn adequately supported by the results?

Partly

Are sufficient details of methods and analysis provided to allow replication by others?

Yes

Reviewer Expertise:

UĞUR DEMİR received the Ph.D. degree in mechatronics engineering from Marmara University, in 2018. He is currently an Associate Professor with the Department of Mechatronics Engineering, Marmara University. He has extensively worked on the development of novel electrical machine technologies and the actuator for electric vehicle traction operations and mechatronics systems. His research interests include the electromagnetic design of electrical machines, design optimization, artificial neural network applications, autonomous driving, and advanced driving technologies.

I confirm that I have read this submission and believe that I have an appropriate level of expertise to confirm that it is of an acceptable scientific standard, however I have significant reservations, as outlined above.

## References

[1] : Improvement of the power to weight ratio for an induction traction motor using design of experiment on neural network. *Electrical Engineering* .2021;103(5) : 10.1007/s00202-020-01204-2 2267-2284 10.1007/s00202-020-01204-2

[2] : Design and optimization of an electromechanical actuator for the latch of a foldable vehicle seat. *Materials Testing* .2020;62(7) : 10.3139/120.111539 749-755 10.3139/120.111539

[3] : Performance assessments of the material for the traction motor cores of an electric racing kart. *Materials Testing* .2021;63(6) : 10.1515/mt-2020-0085 519-528 10.1515/mt-2020-0085

[4] : Multi System Level Driving Scenarious Based Topology Optimization of Bracket Design for 2 DoF Vehicle Simulator. *International Journal of Automotive Science and Technology* .2023;7(3) : 10.30939/ijastech..1244113 248-258 10.30939/ijastech..1244113

[5] : Comparative Design Improvement of the Growing Rod for the Scoliosis Treatment Considering the Mechanical Complications. *IEEE Access* .2023;11: 10.1109/ACCESS.2023.3268147 40107-40120 10.1109/ACCESS.2023.3268147

[6] : Intelligent Design Optimization for Traction and Steering Motors of an Autonomous Electric Shuttle under Driving Scenarios. *Electronics* .2024;13(3) : 10.3390/electronics13030566 10.3390/electronics13030566

# Response

**Published:** 2025-02-12

**Authors:** Borja Lizarribar Carrillo

**Affiliations:** Transport and Energy Division, CEIT, San Sebastián, Basque Country, Spain

We provide below a point by point response to reviewer comments:

**Reviewer: “the results of the study should improve with a comparison of a shelf product which is similar according to the requirements”**

**Author Response:** The additively manufactured design has been done in order to improve an already existing product, and it is lighter comparing to it. However, the topic manager does not allow to share this information of the previous design.

**Reviewer: “ the design optimization techniques for the actuator should be discussed”**

**Author Response: **Thank you for your comment. There are countless design optimisation techniques that are applied to electrical machines, as can be seen in [1]. However, in our experience, the results obtained after the optimisation are not only dependent of the optimisation used but also in the size of population, the initial population… In the case analysed in the paper, a Multi Objective Differential Evolution algorithm has been used due to the simplicity and robustness of this method. Moreover, this optimisation method has been widely used in electrical machine optimisation as it can be seen in [2]-[4]. In order to clarify this aspect, the article ha been modified to highlight the fact that more optimisation methods are available in literature, and the reason why MODE has been used in this study.

**Reviewer: topology optimization is dealt with the design of experiments in terms of the parametric design and objectives.**

**Author Response: **We are sorry but we do not fully understand this remark. In case it is useful, we have optimised parametrically the electromagnetic design, only the active parts of the electric motor, and topology optimisation has been applied only to the mechanical parts, having the electromagnetic function parts frozen in this optimisation. This is explained in the paper in section 2.2 and 2.3.2.

**References** 1 V.S., N., Mahadevan, B., V., K., R., A., Nagarajan, G., S., S. and M., S. (2017), "Design optimization of ferrite assisted synchronous reluctance motor using multi-objective differential evolution algorithm", COMPEL - The international journal for computation and mathematics in electrical and electronic engineering, Vol. 36 No. 1, pp. 219-239. https://doi.org/10.1108/COMPEL-06-2016-0253 2 Alotto, P. (2011), “A hybrid multiobjective differential evolution method for electromagnetic device optimization”, COMPEL: The International Journal for Computation and Mathematics in Electrical and Electronic Engineering, Vol. 30 No. 6, pp. 1815-1828. 3 Cupertino, F., Pellegrino, G. and Gerada, C. (2014), “Design of synchronous reluctance motors with multiobjective optimization algorithms”, IEEE Transactions on Industrial Applications, Vol. 50 No. 6, pp. 3617-3627. 4 Gamba, M., Pelegrino, G. and Cupertino, F. (2014), “Optimal number of rotor parameters for the automatic design of synchronous reluctance machines”, proceedings of the 2014 International Conference on Electrical Machines (ICEM), Berlin, pp. 1334-2134.
